# Small molecule inhibitors reveal PTK6 kinase is not an oncogenic driver in breast cancers

**DOI:** 10.1371/journal.pone.0198374

**Published:** 2018-06-07

**Authors:** Luping Qiu, Kymberly Levine, Ketan S. Gajiwala, Ciarán N. Cronin, Asako Nagata, Eric Johnson, Michelle Kraus, John Tatlock, Robert Kania, Timothy Foley, Shaoxian Sun

**Affiliations:** 1 Center of Therapeutic Innovation, Pfizer Inc., New York, NY, United States of America; 2 Worldwide Medicinal Chemistry, Pfizer Inc., San Diego, CA, United States of America; 3 Primary Pharmacology, Pfizer Inc., Groton, CT, United States of America; Columbia University, UNITED STATES

## Abstract

Protein tyrosine kinase 6 (PTK6, or BRK) is aberrantly expressed in breast cancers, and emerging as an oncogene that promotes tumor cell proliferation, migration and evasion. Both kinase-dependent and -independent functions of PTK6 in driving tumor growth have been described, therefore targeting PTK6 kinase activity by small molecule inhibitors as a therapeutic approach to treat cancers remains to be validated. In this study, we identified novel, potent and selective PTK6 kinase inhibitors as a means to investigate the role of PTK6 kinase activity in breast tumorigenesis. We report here the crystal structures of apo-PTK6 and inhibitor-bound PTK6 complexes, providing the structural basis for small molecule interaction with PTK6. The kinase inhibitors moderately suppress tumor cell growth in 2D and 3D cell cultures. However, the tumor cell growth inhibition shows neither correlation with the PTK6 kinase activity inhibition, nor the total or activated PTK6 protein levels in tumor cells, suggesting that the tumor cell growth is independent of PTK6 kinase activity. Furthermore, in engineered breast tumor cells overexpressing PTK6, the inhibition of PTK6 kinase activity does not parallel the inhibition of tumor cell growth with a >500-fold shift in compound potencies (IC_50_ values). Overall, these findings suggest that the kinase activity of PTK6 does not play a significant role in tumorigenesis, thus providing important evidence against PTK6 kinase as a potential therapeutic target for breast cancer treatment.

## Introduction

Non-receptor protein tyrosine kinase 6 (PTK6, or BRK) is expressed in normal epithelia in the gastrointestinal tract and oral cavity, and regulates cell proliferation and differentiation [1–5]. Aberrant expression of PTK6 is frequently detected in epithelial cancers including breast, ovarian, prostate and colon cancers and linked to tumor formation [3, 6–10]. The association of PTK6 with cancers is widely studied in breast cancers. High transcriptional levels of PTK6 are associated with poor disease prognosis in breast cancers [10–14]. Knockdown of PTK6 expression by shRNA or siRNA in tumor cells leads to significant inhibition of tumor growth, induction of tumor cell apoptosis, and suppression of metastases of triple negative breast cancer, while overexpression of PTK6 promotes cell proliferation [14–18]. A growing body of evidence suggests oncogenic roles for PTK6 in breast cancers, and targeting its kinase activity by small molecule inhibitors has been proposed as a potential therapy for the treatment of breast cancers [11, 19, 20].

Despite the intensive studies of PTK6 function in normal cells and tumor cells, the PTK6-dependent signaling pathways that regulate various cellular processes is poorly understood, and the specific role of PTK6 kinase activity in tumor formation and growth remains unclear. Both kinase-dependent and kinase-independent roles for PTK6 have been described in breast and colon tumors [11, 17, 21]. For example, overexpression of the PTK6 kinase-dead mutant in breast tumor T47D cells promoted cell proliferation at the same level as the PTK6 wild type (WT) protein [17]. Several PTK6 kinase inhibitors have been identified and showed suppression of tumor cell proliferation and the epithelial-mesenchymal transition in breast tumor cells *in vitro* [16, 19, 22–24]. However, small molecule kinase inhibitors are often associated with kinase promiscuity. The broad kinase selectivity of these PTK6 inhibitors is not known, therefore it is not certain whether the observed inhibitory effects on tumor cells is due to the specific inhibition of PTK6 kinase and/or an off-target effect by affecting other kinases.

In this study, a novel chemical class of potent and selective PTK6 inhibitors was identified. Unlike the previously published PTK6 inhibitors that bind to the phosphorylated form of PTK6, namely Type I inhibitors [22, 24], this class of compounds recognizes the unphosphorylated PTK6 (Type II inhibitors), and prevents the activation of PTK6 by stabilizing the inactive form of the enzyme. The crystal structures of apo-PTK6 and PTK6 complexes with both Type I and II inhibitors are described herein, and confirm the different binding modes of inhibitors. PTK6 inhibitors as well as a structural analogue without inhibiting PTK6 kinase were profiled for broad kinase selectivity, and applied to probe the specific role of PTK6 kinase activity in tumor cells. It was found that while PTK6 kinase activity was substantially inhibited by both Type I and II inhibitors in tumor cells, the tumor growth was only weakly suppressed. The inhibition of tumor cell growth by PTK6 kinase inhibitors is independent of PTK6 expression or activation levels in cells, and bears no correlation with the inhibition of PTK6 kinase activity, implying that the observed inhibition of tumor cell growth is not driven by PTK6 kinase inhibition, but rather an off-target effect. These results suggest that PTK6 kinase activity does not play an oncogenic function in human breast cancers.

## Materials and methods

### Chemical compounds

Compounds 21a [22], 21c [22], PF-06698840, PF-06683324 and PF-06737007 were synthesized at Pfizer. All compounds were solubilized in dimethyl sulfoxide (DMSO) before use.

### Cell line

Human breast cancer cell lines MDA-MB-231, MDA-MB-453, T47D, BT474, BT549, human embryonic kidney cell line HEK293T and human breast epithelium cell MCF10A were from ATCC (Manassas, VA). All cell lines were tested negative for mycoplasma contamination. The growth medium for MCF10A was described previously [25]. MDA-MB-453 and T47D cells were grown in RPMI supplemented with 10% FBS. MDA-MB-231 cells were maintained in DMEM supplemented with 10% FBS and non-essential amino acids. All other cell lines were grown in media according to the manufacturer’s instructions.

Stable cell lines HEK293T and MDA-MB-231 overexpressing PTK6 WT were generated using a lentiviral pReceiver-Lv105 expression vector (GeneCopoeia) under a CMV promoter and selected against puromycin according to the manufacturer’s instructions. Overexpression of PTK6 and p-PTK6 in engineered cells was confirmed by Western Blot using anti-PTK6 C18 antibody (Santa Cruz) and anti-p-PTK6 antibody (Millipore), respectively.

### Biochemical kinase assay

The PTK6 biochemical kinase assay was conducted by Carna Biosciences using a mobility shift assay (http://www.carnabio.com/profiling_pdf/p59.pdf). The kinase selectivity of compounds was profiled at 1 uM against a >320-kinase panel by Invitrogen.

### p-PTK6 In-Cell ELISA in engineered cells overexpressing PTK6

The level of auto-phosphorylated PTK6 at Y342 (p-PTK6) in engineered cells was quantified by In-Cell ELISA. In this assay, engineered cells overexpressing PTK6 or its parental control cells were seeded in collagen-coated 96-well plates (Corning) overnight, and then incubated with various concentrations of compounds or 0.1% DMSO control in growth media for 1 hr at 37°C. After being fixed and permeabilized in TBS containing 4% paraformaldehyde and 0.2% Triton X-100 for 20 minutes, cells were blocked with TBS blocking buffer (LI-COR Biosciences) for 1 hour, followed by incubation with anti-p-PTK6 antibody (Millipore, the same antibody used in western blot) in 0.02% Tween and TBS blocking buffer for 2 hours. Cells were washed and then incubated with goat anti-rabbit IR800 (LI-COR Biosciences) and CellTag 700 (LI-COR Biosciences) in 0.02% Tween and TBS blocking buffer according to the manufacturer’s instruction. The fluorescent signal of p-PTK6 was measured in the 800 nm channel by an Odyssey CLx imaging system (LI-COR Biosciences), and normalized by cell numbers measured by Celltag700 stain (Odyssey CLx) in each well, which adjusted for cell plating variation. The p-PTK6 signal in parental control cells was set as baseline. After baseline subtraction, the p-PTK6 level in engineered cells treated with PTK6 inhibitor was expressed as percentage of expression relative to the p-PTK6 level in engineered cells treated with DMSO control. The potency of compound in inhibition of PTK6 kinase activity (IC_50_) was determined by non-linear regression curve fitting to a dose-response equation defined in PRISM (GraphPad Inc.)

### Cytotoxicity assay

The effects of kinase inhibitors on tumor cell growth were assessed in both 2D and 3D cultures. For 2D culture, MDA-MB231, engineered MDA-MB-231 cells overexpressing PTK6 WT, MDA-MB-453, T47D and HEK293T cells were seeded at 2000 cells/well in 96-well plates and incubated with various concentrations of compounds or DMSO control in growth media as described above for 6 days. The final DMSO concentration was 0.1%. Media and compounds were replenished every 2–3 days. The Cell Titer-Glo Luminescent Cell Viability Assay (Promega) was used to quantitate the cytotoxicity of the compounds according to the manufacturer’s instructions.

For 3D culture, 50 uL of growth factor-reduced Matrigel (Corning) was coated in each well of a 96-well plate, followed by seeding 3000 cells/well on top of the matrigel. The rest of the procedure followed the same protocol as the 2D cultures described above.

### Confocal microscopy

Cells were treated with compounds, DMSO or media for 2 hours at 37°C/5% CO_2_ and fixed with 4% para-formaldehyde/PBS (Electron Microscopy Sciences) for 15 minutes at room temperature, followed by permeabilization with 0.1% TritonX100 (Sigma Aldrich) for 5 minutes. Cells were washed with cold PBS, blocked with 5% normal goat serum/PBS for 60 minutes at room temperature and incubated with anti-p-PTK6 antibody (1:50, Millipore, the same antibody used in western blot) and anti-alpha-tubulin antibody (1:100, Sigma Aldrich) in 3% NGS/PBS overnight at 4°C on rocker. Cells were then washed with cold 1% NGS/PBS and incubated with Alexa Fluor 488 and Alexa Fluor 647 (1:500, Thermo Fisher Scientific) secondary antibodies in 3% NGS/PBS for 60 minutes at room temperature, washed as above, and incubated with Hoechst/PBS (1:5000, Thermo Fisher Scientific) for 15 minutes at room temperature. Phosphorylation of PTK6 in cells was imaged using a Leica DMI 4000B laser-scanning confocal microscope.

### Recombinant PTK6 protein production, crystallization and structure determination

The kinase domain (D182-S443) of human PTK6 (Genbank NM_005975.3) was sub-cloned at GenScript into an in-house insect cell expression transfer vector that appended the N-terminus with the TEV-cleavable polyhistidine purification tag sequence MASHHHHHHDYDGATTENLYFQ/GS, where TEV cleaves at the solidus, leaving the recombinant protein with a Gly-Ser extension at the N-terminus. Subsequently, this construct was modified at GenScript to introduce a W184A mutation. Recombinant baculoviruses were prepared by using the Bac-to-Bac method (InVitrogen) and PTK6 was produced in *Sf*21 insect cells. Recombinant PTK6 was purified by affinity chromatography using ProBond resin (Invitrogen) followed by His-tag cleavage by TEV protease. The untagged PTK6 was then further purified by size exclusion chromatography using BioSep S3000 column (Phenomenex) to obtain monomeric PTK6 protein. The protein concentration was determined the Coomassie Plus Protein Reagent (Pierce) using BSA (Pierce) as standard.

PTK6 kinase domain was crystallized in apo form by sitting drop vapor diffusion at 4 ^o^C. The crystallization drops were formed by mixing 100 nL protein solution (11 mg/mL PTK6, 2 mM ATP, 4 mM MgCl_2_, pH 7.4) with 100 nL of crystallization reagent (3.3 M potassium acetate, and 0.1 M bicine pH 8). Co-crystals of the Type I inhibitor 21c were obtained by soaking of compound into apo form crystals. Optimized crystals were obtained by mixing 300 nL protein solution at 10 mg/mL containing 0.9 mM ATP, 4 mM MgCl_2_ and 2.5 mM TCEP, with 700 nL of crystallization reagent by sitting drop vapor diffusion at 13 ^o^C. Compound was added to the crystallization drop to reach a final concentration of ~ 2 mM, and soaking proceeded for 2 hrs before crystal harvest. PTK6 was co-crystallized with the Type II inhibitor PF-6683324 by sitting drop vapor diffusion at 13 ^o^C using 14 mg/mL protein solution in the presence of a 1.5-fold molar excess of compound. The crystallization condition consisted of 1.25 M NaH_2_PO_4_, 0.9 M K_2_HPO_4_, 0.2 M Li_2_SO_4_ and 0.1 M CHES, pH 9.8.

The initial PTK6 structure was solved by using a homology model of the kinase domain as the search model. The homology model was built by using the PDB entries 1FMK (Src) and 4XCU (FGFR4) as the starting templates. Molecular replacement was conducted by using PHASER [26] and the structure was refined using CNX, with the last round of refinement completed by using BUSTER. The refined structure served as the starting model for subsequent structure solutions.

## Results

### Identification of novel, potent and selective PTK6 Type II inhibitors

Previously, Zeng et al [22] reported Type I PTK6 inhibitors that recognize the active form of PTK6 (p-Y342 PTK6). Here, we identified novel, potent and selective Type II PTK6 inhibitors represented by PF-6683324 and PF-6689840 that bind to unphosphorylated PTK6. The different binding modes were confirmed by the co-crystal structures of PTK6 with inhibitors (see below). In addition, PF-6737007, a close structural analogue of PF-6683324 and PF-6689840, was identified as a negative control compound with little activity against PTK6 (IC_50_ >10 uM). Both Type I ([22] and Type II inhibitors as well as PF-6737007 were included in the study to investigate the role of PTK6 kinase activity in tumor cell growth. The compound potencies in inhibition of PTK6 kinase activity are summarized in Table 1. The compound cellular potency of PTK6 kinase inhibition was assessed in engineered HET293T cells overexpressing PTK6 WT by measuring PTK6 phosphorylation at Y342, a PTK6-specific autophosphorylation site in the activation loop [27].

**Table 1:** Compound potency in biochemical kinase assay and p-PTK6 in-cell ELISA assay in engineered HEK293T cells overexpressing PTK6 WT.

Compound (type of inhibitor)	Biochemical assay IC_50_ (uM)	p-PTK6 in-cell ELISA IC_50_ (uM)
**21c (Type I)**	**0.056 ± 0.004**	**0.063 ± 0.018**
**21a (Type I)**	**0.025 ± 0.003**	**0.023 ± 0.011**
**PF-6683324 (Type II)**	**0.076 ± 0.001**	**0.70 ± 0.33**
**PF-6698840 (Type II)**	**0.054 ± 0.001**	**0.25 ± 0.10**
**PF-6737007** **(negative control)**	**>10**	**>10**

The IC_50_ values are listed as the mean ± standard deviation of at least four independent experiments.

**Fig 1:**
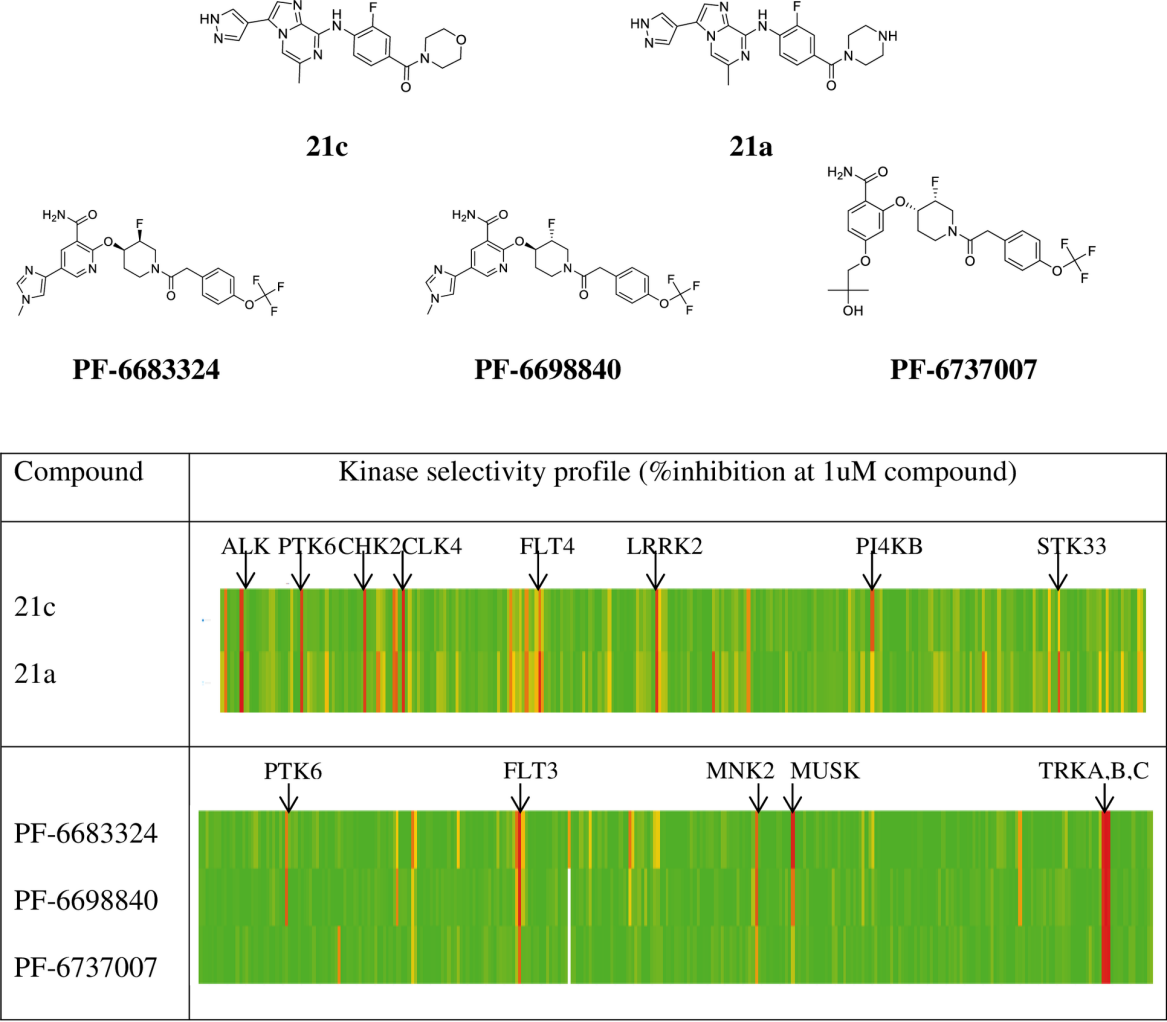
Kinase selectivity of PTK6 inhibitors against more than 320 kinases. The % inhibition is calculated based on the biochemical kinase activity measured in the presence of 1uM compound relative to DMSO control. The color bar represents the level of inhibition: red, 100%; yellow, 50%; and green, 0% inhibition. Compounds 21a and 21c are Type I inhibitors that bind to the active form of PTK6; while PF-6683324 and PF-6698840 are Type II inhibitors that bind to the inactive form of PTK6. PF-6737007 is a structural analogue of PF-6698840 that shares similar kinase selectivity but does not inhibit PTK6 kinase activity. Kinases that are >80% inhibited by compounds at 1uM are marked in the table.

To evaluate a compound’s off target effects, the kinase selectivity of each compound was assessed in a >320-kinase panel covering broad kinase classes (Fig 1). The Type I inhibitors (21a and 21c) and Type II inhibitors (PF-6683324 and PF-6689840) have distinct chemical structures and exhibit non-overlapping kinase selectivity profiles, while compounds within the same chemical series share similar kinase selectivity. The Type II inhibitors tested here appear to have superior kinase selectivity compared to the Type I inhibitors (~3% vs. ~6% of kinases in the panel inhibited >50% at 1uM compound). With exception of PTK6, PF-6737007 displays nearly identical kinase selectivity to its structural analogues PF-6689840 and PF-6683324.

### Crystal structures of apo-PTK6 and inhibitor-bound PTK6 complexes

Numerous full-length and kinase domain constructs of PTK6 were explored in protein crystallization. The protein construct that crystallized included residues 182–443 of the kinase domain with a single W184A mutation. Interestingly, the analogous construct with the wild-type sequence failed to yield crystals. Notably, previously reported structures of PTK6 kinase domain [28] were obtained by using the protein sequence beginning at residue 185. It has been suggested previously that PTK6 harboring the W184A mutation lacks kinase activity [29]; however, in the current study this protein was capable of auto-phosphorylation (data not shown).

**Fig 2:**
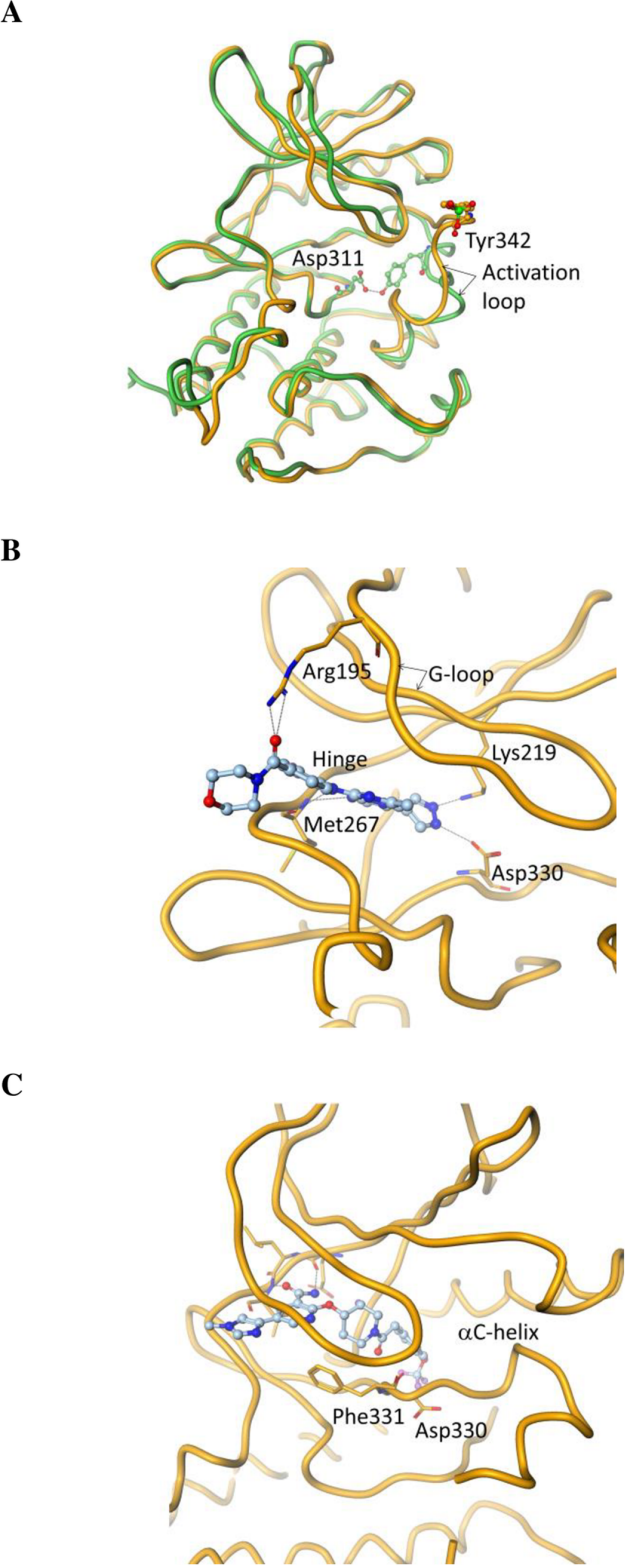
Crystal structures of apo-PTK6 and PTK6 complexes with inhibitors. (A) Apo-PTK6 crystal structure (shown in orange) with Y342 phosphorylated overlaid with the published structure by Thakur et al (28) (shown in green); (B) Active site structure of PTK6 in complex with the Type I inhibitor 21c; (C) Active site structure of PTK6 in complex with the Type II inhibitor PF-6683324. Asp330 and Phe331 belong to the DFG-motif of PTK6.

The apo-PTK6 was crystallized in the presence of ATP and MgCl_2_. The resulting 2.5 Å resolution structure is similar to the previously reported PTK6 structure (PDBID: 5D7V) [28] except for the conformation of the activation loop. Although both apo-structures are in the DFG-in state, the two structures show substantially different conformations of the activation loop downstream of the DFG-motif, likely due to the phosphorylation state of Y342 (Fig 2A). The previously published structure shows part of the activation loop in a distinctly helical state that entirely buries Y342 in the hydrophobic core, where Y342 is in a tight specific polar interaction with D312 of the HRD motif of the catalytic loop. In the apo-structure reported here, the activation loop is mostly a random coil, and Y342 is entirely solvent exposed. We conjecture that the observed activation loop conformation may be the consequence of the phosphorylation of Y342.

The co-crystals of PTK6 with the Type I inhibitor 21c [30] were obtained by soaking the apo-protein crystals with the compound. The resulting 1.8 Å resolution structure is nearly identical in its protein conformation to the apo-protein structure described above. The interactions between the ATP-binding site of the protein and the compound are highlighted in Fig 2B. The aminoimidazopyrizine core of the inhibitor makes two hydrogen bonds with the backbone amides of M267 in the hinge region of the kinase. The pyrazole nitrogen atoms are involved in specific interactions with D330 from the DFG-motif and the catalytically important K219. R195 from the G-loop is engaged in a H-bonding interaction with the carbonyl O of the ligand.

The complex of PTK6 kinase domain with Type II inhibitor PF-6683324 was obtained by co-crystallization and yielded a 1.5 Å resolution crystal structure (Fig 2C). As expected for a Type II inhibitor, the kinase domain is in the DFG-out conformation. However, downstream of the DFG-motif, the activation loop has the same partially helical conformation with Y342 buried as in the previously reported structure of PTK6 (PDBID: 5D7V). The DFG-out conformation accommodates the trifluoromethoxy group of the ligand in the deep hydrophobic core of the protein between the DFG-motif and the αC-helix, which would otherwise be occupied by F331 of the DFG motif in its DFG-in state. The amide group of the ligand is involved in hydrogen bonding interactions with the backbone of the kinase hinge region. The pyrazole is involved in water-mediated interactions with the protein. The N-lobe of the kinase undergoes substantial rigid body motion to open the ATP-binding cleft between the two lobes in order to accommodate the bulky type II inhibitor. The crystallization conditions, data collection and refinement statistics are summarized in Table 2. The structures have been deposited in the database and the PDB codes (PDBID) are listed in Table 2.

**Table 2:** Crystallilzation conditions, data collection and refinement statistics.

	Apo	21c complex	PF-6683324 complex
Crystallization conditions	3.3 M Potassium acetate, 0.1 M bicine, pH 8, 13°C	3.4 M Potassium acetate, 0.1 M bicine, pH 7.5, 13°C	1.25 M Sodium dihydrogen phosphate, 0.89 M di-Potassium hydrogen phosphate, 0.2 M Lithium sulfate, 0.1 M CHES, pH 9.8, 13^o^ C
Data collection			
Beamline	APS 17ID	APS 17ID	APS 17ID
Resolution	2.5	1.8	1.5
Space group	R3	R3	P2_1_2_1_2_1_
Unit cell parameters (Å)	108.6, 108.6, 84	107.9, 107.9, 84.8	37.9, 54.9, 134.6
Measured reflections	66,464	197,238	275,953
Unique reflections	13,913	34,192	46,820
Data redundancy	4.8	5.8	5.9
Data completeness (%)	98.7 (100)	99.9 (100)	97.9 (86.8)
R_merge_	0.103 (1.026)	0.039 (0.468)	0.037 (0.418)
R_pim_	0.053 (0.521)	0.018 (0.215)	0.016 (0.239)
CC_1/2_	0.997 (0.763)	1.0 (0.924)	0.999 (0.903)
I/σ	11.3 (2.5)	24.3 (3.7)	22.9 (2.6)
Refinement			
R_work_/R_free_ (%)	20.43/24.7	19.77/21.6	20.3/20.95
Number of protein atoms	2,127	2,188	2,109
Number of non-protein molecules	24 water	112 water 1 ligand	180 water 1 ligand
RMSD, bond lengths (Å)	0.007	0.005	0.004
RMSD, bond angles (^o^)	1.1	0.9	0.8
Ramachandran plot (favored/allowed) (%)	90.1/9.9	92.2/7.8	92.5/7.5
PDBID	6CZ2	6CZ3	6CZ4

### Inhibition of tumor cell growth by PTK6 inhibitors is independent of PTK6 expression levels in cells

**Fig 3:**
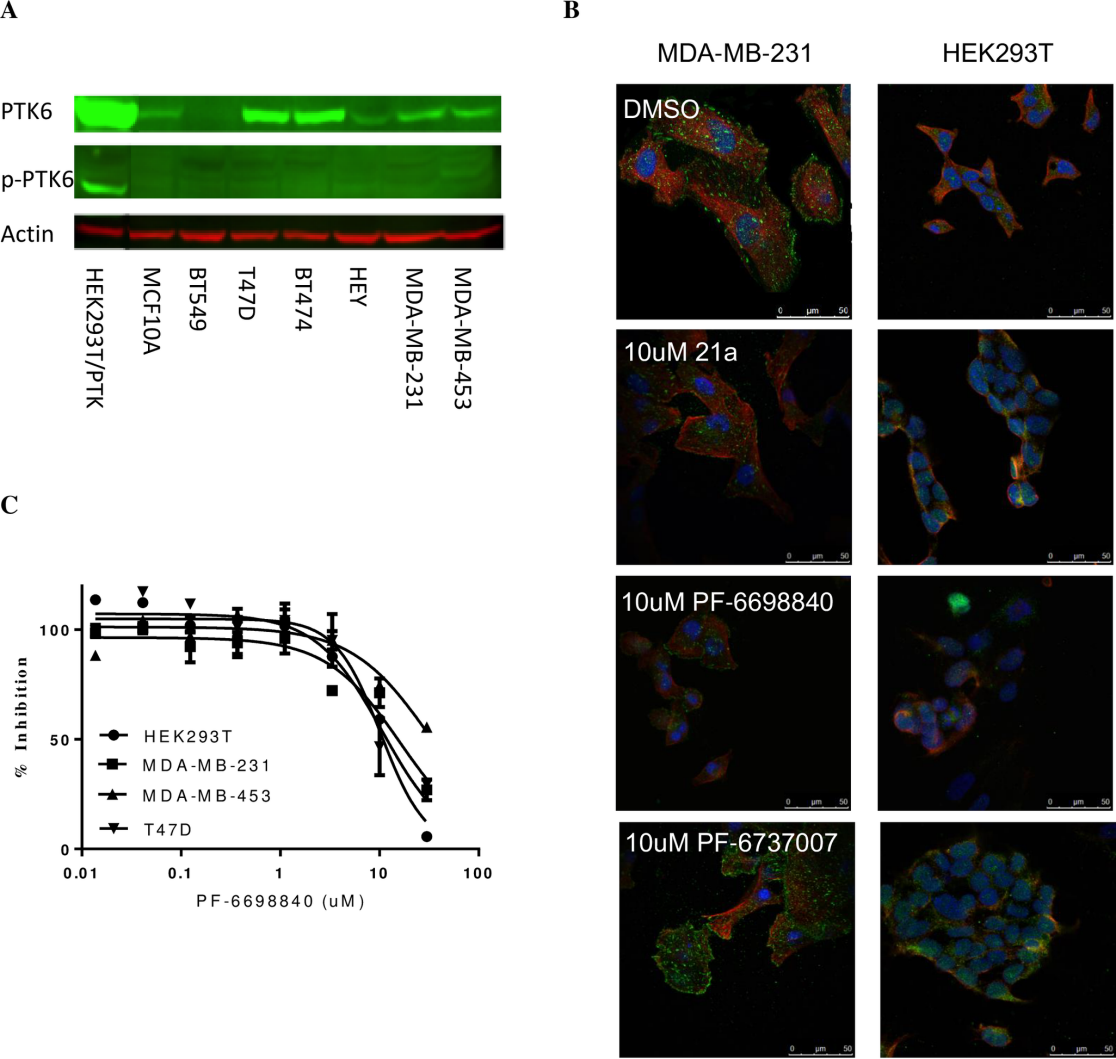
Inhibition of tumor cell growth by Type I and Type II PTK6 inhibitors is independent of PTK6 expression levels in cells. (A) PTK6 expressions and activation (p-Y342 PTK6) in tumor cell lines and breast epithelial cells MCF10A cells analyzed by Western Blot. Engineered HEK293T cells overexpressing PTK6 WT was used as a positive control. (B) Detection of p-Y342 PTK6 in tumor cells MDA-MB-231 by confocal microscopy. Cells were treated with DMSO, 10 uM PTK6 inhibitor 21a, 10 uM PF-6698840 or 10 uM PTK6-negative control compound PF-6737007 for 2 hours. α-Tubulin (ubiquitously expressed in cells) and DAPI (restricted expression in nucleus) are shown in red and blue, respectively. p-PTK6 (green) was detected on the cell membrane of MDA-MB-231 cells. HEK293T cells do not have detectable PTK6 expression and were used as a negative control. (C) Cell growth inhibition by PTK6 inhibitors in PTK6-positive tumor cells and PTK6-negative HEK293T cells. Cells were treated with DMSO or compounds for 6 days in 2D or 7 days in 3D culture, and the cell growth inhibition was measured by Cell Titer-Glo on day 6 or 7.

Various levels of PTK6 protein expression were detected by Western blot in breast tumor cell lines with rank order of T47D = BT474 > MDA-MB-231 = MDA-MB-453 > BT549 (Fig 3A). A modest level of PTK6 was also detected in “normal” breast epithelial cells MCF10A. However, the phosphorylated PTK6 (p-Y342 PTK6) in tumor cell lines was not detected by Western Blot likely due to its low expression levels, instead observed on cell membranes by confocal microscopy, consistent with a recent report (16). As expected, the membrane associated p-PTK6 was diminished by the treatment of PTK6 inhibitor 21a or PF-6698840, whereas unchanged by the negative control compound PF-6737007 (Fig 3B). However, quantitative image analysis of the membrane associated p-PTK6 levels as a function of compound concentrations (S1 Fig) was not successful due to low signal to noise window. Notably, T47D cells that have highest level of total PTK6 have little p-PTK6 detected, whereas MDA-MB-231 cells with lower total PTK6 expression have highest p-PTK6 observed on the cell membrane. So overall the level of PTK6 activation was low and did not mirror the total PTK6 expression in tumor cells.

The effects of PTK6 inhibitors on tumor cell growth were measured in a number of cell lines with various PTK6 expression levels. The cell growth was moderately suppressed by PTK6 inhibitors in all cells tested, including HEK293T cells which have no detectable PTK6 expression. It appears that the sensitivity of tumor cell growth to PTK inhibitors has no correlation to the PTK6 total expression or activation levels in the cells, suggesting tumor cell growth is not driven by PTK6 kinase activity. The potencies (IC_50_) of both Type I and Type II inhibitors in inhibition of cell growth are summarized in Table 3, and representative dose-dependent inhibition curves are shown in Fig 3C.

**Table 3:** Cell growth inhibition by PTK6 inhibitors in PTK6-positive tumor cells and PTK6-negative HEK293T cells.

Cell line	PTK6/p-PTK6 expression	21a, IC_50_ (uM)		PF-6698840, IC_50_ (uM)	
		3D culture	2D culture	3D culture	2D culture
HEK293T	-/-	6.1 ± 3.1	7.3 ± 3.2	17 ± 10	17 ± 10
MDA-MB-231	+/++	15 ± 9	18 ± 9	>10	>10
MDA-MB-453	+/+	6.4 ± 2.4	6.6 ± 3.4	>20	>20
T47D	++/+	>10	>10	9 ± 3	7

The compound potency IC_50_ was determined by non-linear regression curve fitting to a dose-response equation in Graphpad Prism. The IC_50_ values were similar in 2D and 3D cultures and described as the average ± standard deviation.

The relative expression levels of PTK6 and p-PTK6 in cells are indicated with ++ as highest level, and–as undetectable level.

### Inhibition of tumor cell growth by PTK6 inhibitors is independent of PTK6 kinase activity inhibition

**Fig 4:**
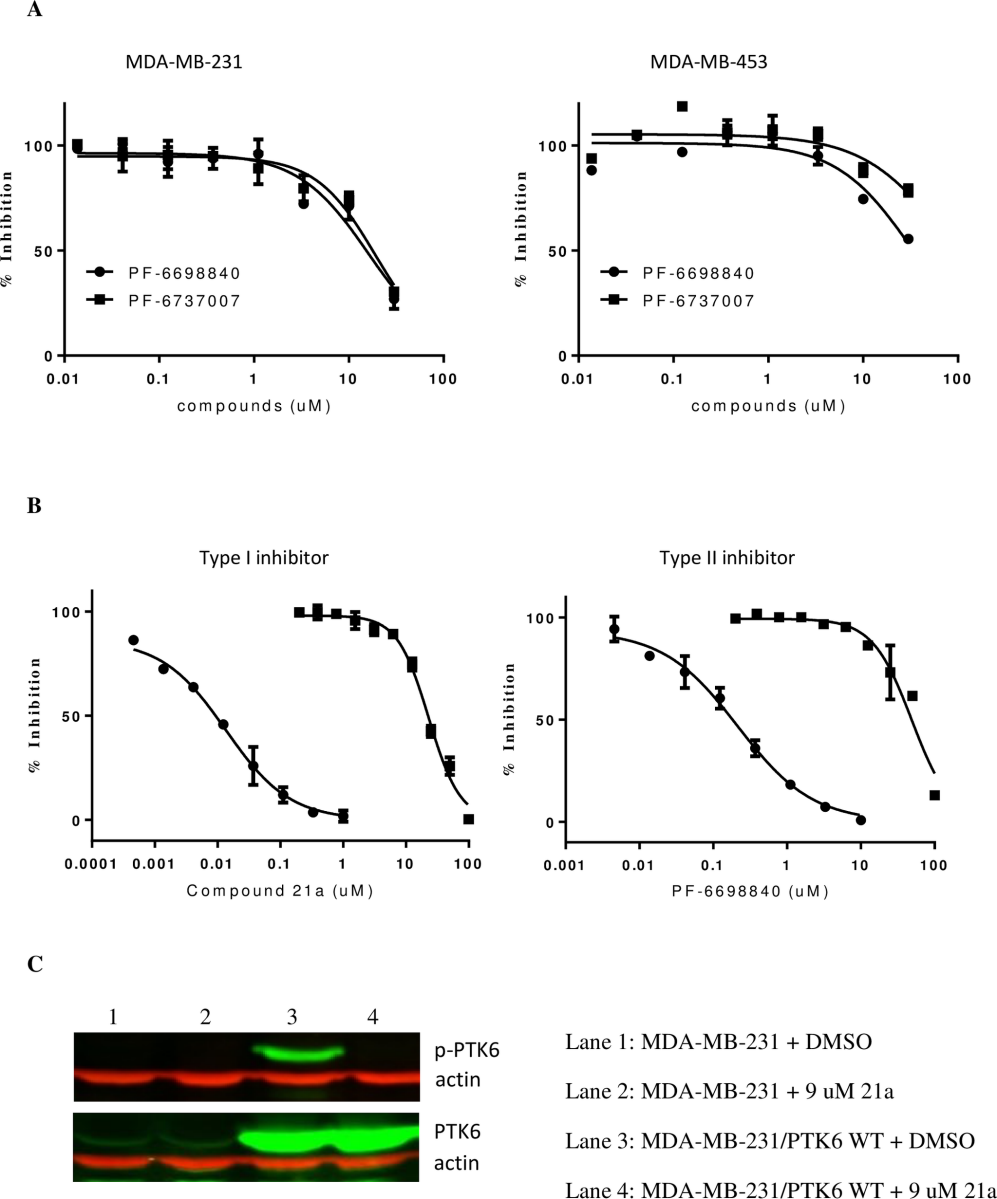
Inhibition of tumor cell growth by PTK6 inhibitors is independent of PTK6 kinase activity inhibition. (A) Tumor cell growth inhibition by PTK6 inhibitor PF-6698840 (circle) and PTK6 negative control compound PF-6737007 (square) in breast tumor cell lines MDA-MB-231 and MDA-MB-453. Similar potencies were observed in the tumor growth inhibition for both compounds despite their significant difference in the potency of inhibiting PTK6 kinase activity; (B) Dose dependent inhibition of p-PTK6 kinase activity (circle) and cell proliferation (square) by Type I inhibitor 21a and Type II inhibitor PF-6698840 in engineered MDA-MB-231 cells overexpressing PTK6 WT. The p-PTK6 kinase activity was assessed by In-Cell ELISA in which cellular levels of autophosphorylation of PTK6 at Y342 were measured by fluorescence signal (OdysseyCLx imager, LI-COR Bioscience) using anti-p-PTK6 antibody and fluorescent dye–labeled detection reagents. The cell proliferation was measured by Cell Titer-Glo over the course of 6 days. The data was normalized in reference to DMSO control and expressed as percentage of inhibition at various concentrations of inhibitor in the graph. The solid lines represent a non-linear regression curve fit to a dose-response equation defined in PRISM (Graphpad Inc.); (C) Western Blot analysis of p-PTK6 and total PTK6 in the engineered MDA-MB-231 cells in the presence and absence of PTK6 inhibitor. The level of p-PTK6 in engineered MDA-MB-231 cells overexpressing PTK6 WT was diminished by 1 hour treatment of PTK6 inhibitor 21a at 37°C (upper lane 4, treated with 21a vs. upper lane 3, treated with DMSO), while total PTK6 remained unchanged (lower lane 4 treated with 21a vs. lower lane 3 treated with DMSO). Lanes 1–2 represent parental MDA-MB-231 cells, in which p-PTK6 was not detected by western blot due to low level of expression.

Due to the quantification limitation of low endogenous p-PTK6 levels in tumor cells, we were not able to directly measure the PTK6 kinase activity in correlation to the tumor cell growth. Instead, we took the approach of applying PTK6 kinase inhibitor PF-6698840 in comparison to its close structural analogue PF-6737007 lacking PTK6 activity. Since these two compounds share similar off-target kinase selectivity, it provides a tool to investigate specifically the role of PTK6 kinase activity in tumor cell growth. Treatment of breast tumor cells MDA-MB-231 and MDA-MB-453 with either compound leads to modest cell growth inhibition with similar potency despite their significant differences in PTK6 kinase inhibition (Fig 4A), suggesting that their off-target effects may account for the observed tumor growth inhibition. To further confirm the disconnect between PTK6 kinase activity and tumor growth, we overexpressed PTK6 WT in MDA-MB-231 cells, and compared the compound potency of PTK6 kinase inhibition vs. tumor growth inhibition in the engineered MDA-MB-231 cells (Fig 4B). While the compounds robustly inhibit the PTK6 kinase activity with IC_50_ = 0.016 ± 0.007 uM for 21a, and 0.12 ± 0.06 uM for PF-6698840, their inhibition on tumor cell growth was weak with at least 400-fold shift in IC_50_s, suggesting that the inhibition of PTK6 kinase activity does not translate to tumor cell growth suppression.

## Discussion

The diverse roles of PTK6 in signaling pathways that regulate cellular proliferation, differentiation, migration and wound healing [11, 18, 20] may be contributed to its flexible subcellular locations and a divergent pattern of recognition motifs in its SH2 and SH3 domains that mediate interactions with a variety of proteins. More than 30 PTK6 interacting proteins have been identified *in vitro* [11], however, not all of them are PTK6 kinase substrates, suggesting a kinase-independent role of PTK6 as a protein adaptor. Most of studies that suggest an oncogenic role of PTK6 were conducted by either disruption of PTK6 gene or overexpression of PTK6 protein [15, 21, 31]. However, the specific role of PTK6 kinase-dependent *vs*. -independent activity contributing to its oncogenic function has not been clearly elucidated. Biochemical analysis of cancer-associated PTK6 mutations has revealed that the mutants had either increased or decreased kinase activity compared to the wild type protein [32], reflecting a complex regulatory mechanism of PTK6 involvement in tumorigenesis, rather than a strictly kinase activity-driven phenotype. The goal of this study was to determine if PTK6 kinase activity plays a significant oncogenic role using small molecule kinase inhibitors as a probe.

Several small molecule PTK6 kinase inhibitors were previously investigated for their anti-tumor effects in tumor models [16, 19, 22]. However, the most common challenge of using small molecule kinase inhibitors in validating a kinase role is the compound promiscuity in kinase selectivity due to the highly conserved nature of the ATP binding pocket among kinases. It is not surprising kinase inhibitors affect cell growth by hitting multiple kinases, which can lead to misinterpretation of target vs. off-target effects. To overcome this challenge, we took multiple approaches. First, Type II PTK6 inhibitors were identified that recognize the inactive form of the enzyme with improved PTK6 selectivity compared to the published Type I inhibitors [22, 24]. Type II kinase inhibitors generally offer greater selectivity due to their reach into the specific hydrophobic core of the protein behind the ATP-binding site. Second, a structural analogue of the Type II inhibitors was identified that possessed nearly identical kinase selectivity but lacked PTK6 inhibition as a negative control compound. This allowed for the differentiation of PTK6-specific effects from non-specific effects (off-target). Third, both chemically diverse Type I and II inhibitors with non-overlapping kinase selectivity were applied to rule out any chemical structure-biased pharmacological effects or mechanism-dependent inhibitory effects.

From this study, a moderate tumor cell growth inhibition by PTK6 inhibitors was observed. However, there was no differentiation in tumor cell growth inhibition between a potent PTK6 kinase inhibitor and a PTK6-negative control compound, nor a correlation between the sensitivity of tumor cells to PTK6 inhibitors and the PTK6 expression or activation levels, therefore arguing against an oncogenic role of PTK6 kinase activity in tumor cells suggested by other reports (16, 19). The observed anti-tumor effects by PTK6 kinase inhibitors may be caused by off-target kinase inhibition. The significant IC_50_ shift between the PTK6 kinase inhibition and tumor cell growth inhibition in engineered tumor cells overexpressing PTK6 further confirmed the conclusion that PTK6 kinase inhibition does not lead to tumor cell growth suppression.

We also investigated several reported PTK6 substrate candidates that link PTK6 kinase to oncogenic signaling in tumor cells, including Akt [33], Paxillin [34], STAT3/5 [35, 36], ERK1/2 [37], p38 [18], FAK [38] and p130CAS [39]. The phosphorylation of these substrate candidates induced by EGF stimulation (a known PTK6 stimulus) remained unchanged following treatment with PTK6 inhibitors in breast tumor cells MDA-MB-231 and HCC1954 (data not shown), casting a doubt if they are physiological PTK6 substrates in tumor cells. Most of the reported PTK6 substrate candidates were identified from engineered cell lines overexpressing PTK6. Ectopic expression of PTK6 in engineered cells may have a different intracellular location from the endogenous PTK6 in tumor cells given the known flexibility of the intracellular location of PTK6 in different cell types. For instance, the overexpression of PTK6 WT in HEK293T cells is found predominately in nuclei, whereas in engineered MCF10A cells, it is distributed in cytoplasm, nuclei and membranes. In contrast, the endogenous p-PTK6 in tumor cells is observed mostly on cell membranes. This cell type-dependent spatial distribution of PTK6 in engineered cell lines may account for the poor translation of PTK6 substrate candidates identified from engineered cell lines to tumor cells. Overall, we were unable to confirm the reported PTK6 kinase-dependent oncogenic signaling pathways.

These findings do not rule out the possibility that PTK6 may play a role in tumor growth and invasion through a kinase activity-independent function, such as a scaffolding role with other protein partners involved in tumorigenesis. While some evidence suggests an oncogenic function is associated with the membrane localization of PTK6 [10, 40], further investigation is required to determine whether the subcellular translocation of PTK6 drives malignant transformation, and the regulatory mechanism that triggers the localization event and subsequent signaling.

In conclusion, novel, potent and selective PTK6 inhibitors and a negative control analogue have been identified to enable a thorough interrogation of the specific role of PTK6 kinase activity in tumor cells. It is concluded that PTK6 kinase activity does not drive an oncogenic function, therefore targeting PTK6 kinase activity is unlikely to be an effective therapeutic approach to treat breast cancers. The involvement of PTK6 in tumor growth and invasion through a kinase activity-independent function remains to be further investigated.

## Supporting information

S1 Fig Detection of p-Y342 PTK6 in tumor cells MDA-MB-231 treated with PTK6 inhibitor 21a by confocal microscopy. Cells were treated with DMSO or various concentrations of 21a for 2 hours. α-Tubulin (ubiquitously expressed in cells) and DAPI (restricted expression in nucleus) are shown in red and blue, respectively. p-PTK6 (green) was detected on the cell membrane of MDA-MB-231 cells.
(DOCX)
