# Supplementary material for: Small molecule inhibitors reveal PTK6 kinase is not an oncogenic driver in breast cancers
Source: PLoS One. 2018 Jun 7;13(6):e0198374. doi: 10.1371/journal.pone.0198374 (PMC5991704; doi:10.1371/journal.pone.0198374)
Supplement: S1 Fig — Cells were treated with DMSO or various concentrations of 21a for 2 hours. α-Tubulin (ubiquitously expressed in cells) and DAPI (restricted expression in nucleus) are shown in red and blue, respectively. p-PTK6 (green) was detected on the cell membrane of MDA-MB-231 cells. (DOCX) [file pone.0198374.s001.docx]

S1 Fig.

**10uM 21a**

**1uM 21a**
